# Southern Students in Northern Medical Colleges

**Published:** 1859-12

**Authors:** 


*Southern Students in Northern Medical Colleges.*—As our printer is closing the last editorial matter, dispatches come to our community, stating that Southern students of medicine are leaving Philadelphia for the South in large numbers; and the same dispatches contain an indirect charge against the medical schools of the South, of complicity in this unhappy affair. It is scarcely necessary that we should notice the baseless slander, and we would not do so, except under circumstances such as now excite our people throughout the land. For the New Orleans School of Medicine, then, we repel the charge. This school has met with signal success as the result of fair and honorable dealing, and even if she were not thoroughly imbued with a correct idea of moral right and wrong, she could have no interest in inciting our Southern youths at the North to leave the institutions to which they have attached themselves.

But while we were writing the above, here comes a telegraphic dispatch from Southern students in a Northern medical college, expressing their determination to leave, and asking our faculty on what terms they will receive them. They have paid for the lectures North, but they will not remain where they are.—The faculty have duly considered the extraordinary condition of these young men, they regret as sincerely as men can do the state of affairs, but they cannot close their doors on them, and they have told them that they can finish their course in the New Orleans School of Medicine by paying the matriculation fee, and if any desire to graduate, such must pay the necessary fee.

Once more we say, nothing but sincere regret must be felt by every good man, that such events are occurring in the schools of the North, for it evinces a lamentable difference of opinion on vital questions concerning our country's welfare, before which consideration all others sink into insignificance. We deplore it, and we wish from the bottom of our hearts that young men who have attached themselves to Northern schools this season could be content to remain and receive that for which they have paid, and which will be so well bestowed on them; but if they cannot, we cannot say, our doors are closed on you because of your honest convictions.—*N. O. Medical News and Hospital Gazette.*

